# Exome Sequencing Reveals Genetic Variability and Identifies Chronic Prognostic Loci in Chinese Sarcoidosis Patients

**DOI:** 10.3389/fonc.2022.910227

**Published:** 2022-07-04

**Authors:** Qian Zhang, Hui Huang, Meijun Zhang, Chuling Fang, Na Wang, Xiaoyan Jing, Jian Guo, Wei Sun, Xiaoyu Yang, Zuojun Xu

**Affiliations:** ^1^ Department of Respiratory Medicine, Peking Union Medical College Hospital, Chinese Academy of Medical Sciences & Peking Union Medical College, Beijing, China; ^2^ ANNOROAD Co., Beijing, China

**Keywords:** whole-exome sequencing, sarcoidosis, non-synonymous mutations, adaptive immune response, chronic sarcoidosis prognosis

## Abstract

**Background:**

Sarcoidosis is an inflammatory disease characterized by non-caseating granuloma formation in various organs, with several recognized genetic and environmental risk factors. Despite substantial progress, the genetic determinants associated with its prognosis remain largely unknown.

**Objectives:**

This study aimed to identify the genetic changes involved in sarcoidosis and evaluate their clinical relevance.

**Methods:**

We performed whole-exome sequencing (WES) in 116 sporadic sarcoidosis patients (acute sarcoidosis patients, n=58; chronic sarcoidosis patients, n=58). In addition, 208 healthy controls were selected from 1000 G East Asian population data. To identify genes enriched in sarcoidosis, Fisher exact tests were performed. The identified genes were included for further pathway analysis using Gene Ontology (GO) and the Kyoto Encyclopedia of Genes and Genomes (KEGG). Additionally, we used the STRING database to construct a protein network of rare variants and Cytoscape to identify hub genes of signaling pathways.

**Results:**

WES and Fisher’s exact test identified 1,311 variants in 439 protein-coding genes. A total of 135 single nucleotide polymorphisms (SNPs) on 30 protein-coding genes involved in the immunological process based on the GO and KEGG enrichment analysis. Pathway enrichment analysis showed osteoclast differentiation and cytokine–cytokine receptor interactions. Three missense mutations (rs76740888, rs149664918, and rs78251590) in two genes (PRSS3 and CNN2) of immune-related genes showed significantly different mutation frequencies between the disease group and healthy controls. The correlation of genetic abnormalities with clinical outcomes using multivariate analysis of the clinical features and mutation loci showed that the missense variant (rs76740888, Chr9:33796673 G>A) of PRSS3 [*p*=0.04, odds ratio (OR) = 2.49] was significantly associated with chronic disease prognosis. Additionally, the top two hub genes were CCL4 and CXCR4 based on protein–protein interaction (PPI) network analysis.

**Conclusion:**

Our study provides new insights into the molecular pathogenesis of sarcoidosis and identifies novel genetic alterations in this disease, especially PRSS3, which may be promising targets for future therapeutic strategies for chronic sarcoidosis.

## Introduction

Sarcoidosis (MIM 609464) is an immune-mediated disease affecting multiple organs and is characterized by non-caseating necrotizing granulomatous lesions with an elusive etiology (1). The disease is characterized variably across races, and the prognosis and course of the disease depend on the phenotypic characteristics. Compared to white Americans (10.9/100,000), African Americans (35.5/100,000) are affected more frequently, and African Americans tend to develop chronic, severe disease prognoses (2, 3). Some patients may experience spontaneous remission, but others may suffer from a chronic course, ultimately leading to death in severe cases (4). Epidemiological studies suggest that the disease has a racial predisposition and family clustering characteristics (5). Most researchers agree that the etiology of sarcoidosis is due to environmental exposure, genetic factors, and immune system dysregulation. Sarcoidosis is a multiple-gene-affected disease; many published studies have reported the candidate genes of sarcoidosis. Genetic predisposition plays a vital role in the etiology of sarcoidosis and contributes to the heterogeneity of clinical manifestations and prognosis (6).

The classification of sarcoidosis prognosis is based on the duration of the disease course: acute sarcoidosis (≤2 years) and chronic sarcoidosis (>2 years) (7). Up to 40% of patients develop chronic disease with persistent lung inflammation and tissue fibrosis, which contribute to the majority of sarcoidosis mortality (8). Several genetic mutations have been associated with the clinical course of sarcoidosis, and various distinct ethnic groups argue for genetic influence (3). Previous studies suggested that class II HLA-DRB1*03:01 is associated with resolving disease more than the persistent group in Finnish, Croatia, and Czech sarcoidosis patients (9–11). Other non-HLA gene polymorphisms associated with clinical course, including TLR3 (L412F, rs3775291), promoted a persistent clinical phenotype in Irish and American Caucasian patients (12); also, tumor necrosis factor-β (TNF-β) alleles TNF-β1 and TNF-β3 were found to be associated with prolonged clinical course in Japan and Dutch sarcoidosis patients, respectively (12, 13).

In addition to classical candidate-gene filtering methods, genome-wide association analysis (GWAS) also contributes to identifying suspected genes associated with disease etiology. Hofmann et al. reported using GWAS to identify the ANXA11 gene as a new susceptibility locus for sarcoidosis from over 440,000 single nucleotide polymorphisms (SNPs) among 500 patients and controls (14). Additionally, Franke et al. found that the C10ORF67 gene was significantly associated with sarcoidosis and Crohn’s disease among over 83,000 SNPs using the GWAS method (15). Using the whole-exome sequencing method, Elisa Lahtela et al. reported that variations in AADACL3 and C1orf158, located on chromosome 1p36.21, were associated with resolved disease prognosis among 72 Finnish patients (16).

However, limited studies have reported the association between genetic markers and chronic sarcoidosis prognosis in the Chinese population, which requires in-depth research to diagnose and treat sarcoidosis. We present a strategy using whole-exome sequencing data of sarcoidosis patients to evaluate which genetic variants distinguish chronic sarcoidosis prognosis. We identified sequence variations in a sample of 116 Chinese sarcoidosis cases, acute and chronic prognosis, to pinpoint the genetic variety of sarcoidosis prognosis.

## Materials and Methods

### Study Population

One hundred sixteen sarcoidosis patients who were consecutive cases from January 2016 to December 2017 in Peking Union Medical College Hospital and 208 healthy controls were selected for the whole-exome sequencing (WES) study. The patients who underwent WES were diagnosed based on the American Thoracic Society (ATS)/European Respiratory Society (ERS)/World Association of Sarcoidosis and Other Granulomatous Disorders (WASOG) criteria (17). The inclusion criteria included clinical manifestation, radiological characteristics, and pathological evidence. The stages of sarcoidosis were determined following the “Scadding” classification for sarcoidosis. Radiological evaluation of sarcoidosis in the outpatient clinic at Peking Union Medical College Hospital was performed by two physicians with expertise in the respiratory department. All patients who had a clinical follow-up of at least 4 years participated. The diagnosis of the patients with sarcoidosis was confirmed by transbronchial lung biopsy (TBLB). The clinical outcomes of sarcoidosis patients were classified into the acute group (resolve within 2 years, n=58 patients, 50%) and the chronic group (persisting over 2 years, n=58 patients, 50%) (17). All resources were investigated in the Electronic Health Record database of the Peking Union Medical College Hospital. This study was conducted in accordance with the Declaration of Helsinki, and the protocol used to collect human blood samples and clinical resources was approved by the Ethics Committee of Peking Union Medical College Hospital. Written informed consent was obtained from all subjects.

### Whole-Exome Sequencing

DNA was extracted from the blood samples using the QIAampTM DNA and Blood Mini Kit (Qiagen, Valencia, CA) according to the manufacturer’s instructions. WES was performed by ANNOROAD Co. (Beijing, China) using the SureSelectXTTarget Enrichment System (G7530-90000) method for exon capture, and a library was constructed. Then, the paired-end sequencing program was run on the Illumina NovaSeq S2 sequencing platform, and 150-bp reads were obtained. CASAVA 1.8 was used to complete imaging analysis and base detection of the high-throughput sequencing image files with data filtering. Burrows–Wheeler Aligner (BWA) v0.7.17 software was used to compare the sequencing results and the human genome reference sequence (UCSC GRCh37/hg19). Then, The Genome Analysis Toolkit (GATK) v3.8 was used to perform variant calling and to identify SNPs and insertions and deletions (InDels).

### Initial Variants and Sample Quality Control

**Figure 1:**
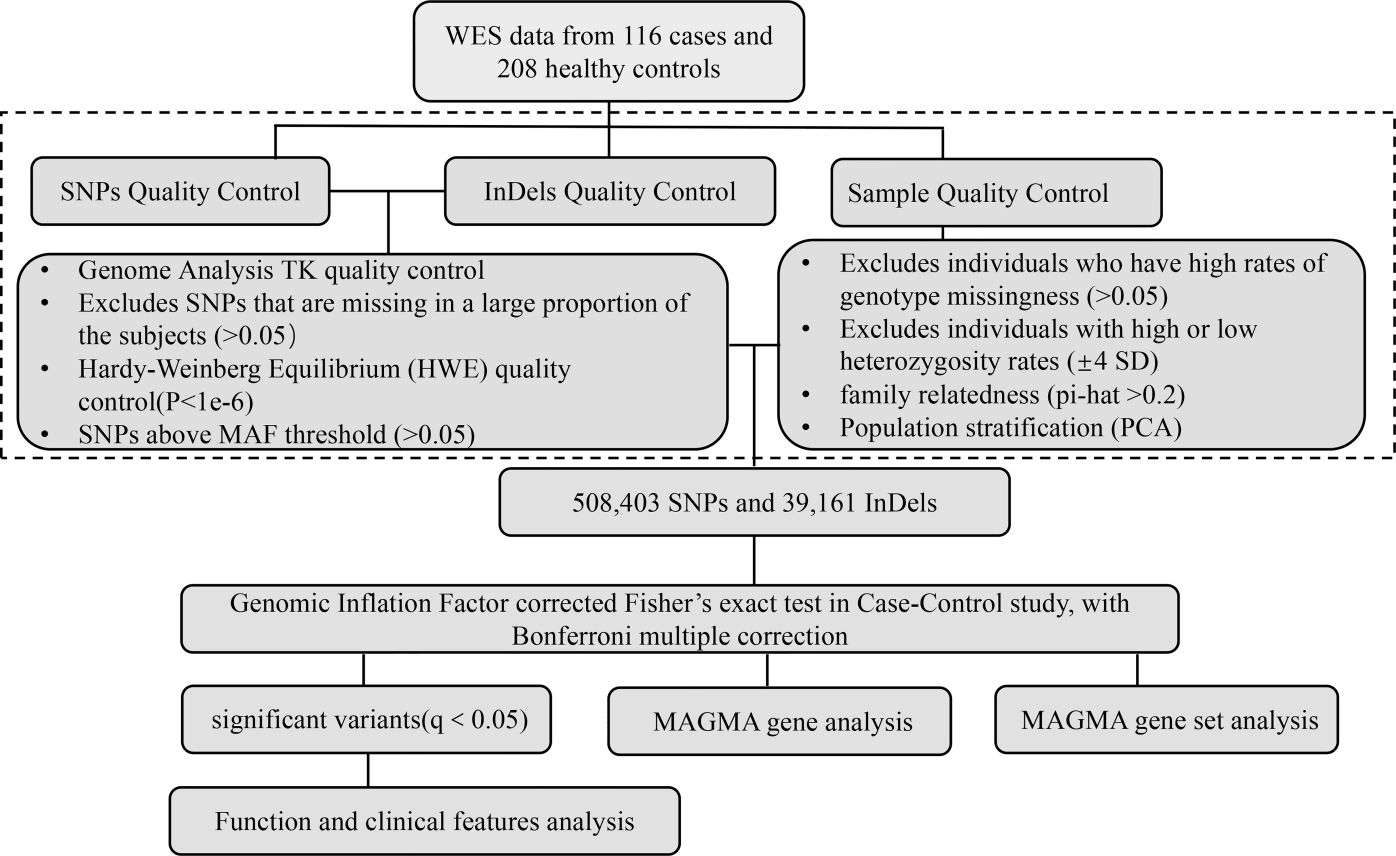
Analytical strategy workflow for variant filtration and candidate gene selection. A schematic overview of the steps involved in whole-exome sequencing analysis with pathogenesis candidate gene detection is shown. SNPs, single nucleotide polymorphisms; InDels, insertions and deletions; WES, whole-exome sequencing; MAF, minor allele frequency; MAGMA, Multimarker Analysis on GenoMic Annotation.

We performed the initial variant and sample quality control (shown in **Figure 1**). All QC steps were analyzed using the software package PLINK v1.09. After variant quality control of the raw data, 5,771,425 SNPs passed SNP quality control with the recommendation from GATK. Among them, 3,981,961 (69%) variants passed Hardy–Weinberg equilibrium (HWE) quality control (*p* < 1e−6). Finally, 508,403 SNPs passed the SNP sample missing rate (<5%) quality control. In addition, 1,223,109 InDels passed quality control with the recommendation from GATK. Of the InDels, 1,000,293 (82%) passed HWE quality control. Additionally, 39,161 InDels passed the sample missingness rate (<5%) quality control. Then, we manipulated the sample quality control and found that all 116 samples and 208 healthy controls passed heterozygosity (mean ± 4 SD), sample missing rate (<5%), and familiar relationships (pi-hat<0.2), meaning that all samples and controls could be used for further evaluation (shown in **Supplementary Figure S1**).

### Mutation Site Filtering and Annotation

The SNPs and InDels identified in 116 sarcoidosis patients were tested with Fisher’s exact test. In this disease research, sample collection was challenging, and therefore, we had a smaller sample size. However, the samples were randomly collected, and to control for false positives, we used different methods to obtain true positive sites and genes. For the Fisher’s exact test, to control population stratification, the genomic inflation factor was used to adjust the chi-2 value and recomputed *p*-value; to control false positives in multiple comparisons, the *p*-values were subjected to Bonferroni multiple corrections (Q-value ≤ 0.05). With Fisher’s exact test results, the Multimarker Analysis on GenoMic Annotation (MAGMA) v1.6 software package (18) was used to perform SNP-wide mean model for gene-based association analysis with the default setting. SNPs were assigned to the genes obtained from Ensembl build 85 (only protein-coding genes). Genome-wide significance was set at 0.05/(the number of tested genes). Genes whose *p*-value reached genome-wide significance can be labeled in the Manhattan plot. Using the result of gene analysis (gene-level *p*-value), gene-set analysis was also performed with default parameters of MAGMA v1.6. The gene sets were obtained from sigdb v7.0 for “Curated gene sets” and “GO terms.” The R package “clusterProfiler” was used to perform Kyoto Encyclopedia of Genes and Genomes (KEGG) enrichment analysis (19). The expression characteristics of the immune-related candidate genes were determined based on the data obtained from Genotype-Tissue Expression (GTEx; gtexportal.org). The characteristics of mutations in immune-related candidate genes were identified using Maftools in the R package (20). The STRING online database (STRING, https://www.string-db.org/) and PPI pairs with a combined score of ≥0.4 were used to construct a PPI network. Cytoscape software v3.7.2 was used to predict the regulatory relationship between genes and analyze the topological parameters of the network. The Genome Reference Consortium Human Build 37 (GRCh37) of *Homo sapiens* in the NCBI database was utilized for SNP description.

### Statistical Analysis

Statistical analyses were performed using SPSS version 26 and GraphPad ® Prism Version 8.0.0 for Mac OS X (San Diego, CA, USA). The normality of the variables was estimated using the Shapiro–Wilk normality test. Non-normally distributed continuous variables were expressed as medians and interquartile ranges [M, (Q1, Q3)], and normally distributed continuous variables were described as the means and standard deviations. Categorical variables are shown as counts and percentages. The independent samples t-test was used for comparing variables with normal distribution. The non-parametric test (Mann–Whitney U test) was used to compare non-normally distributed continuous variables. Pearson’s two test or Fisher’s exact test was used to analyze the categorical variables. Binary logistic regression (backwards method) was used to explore independent factors (age, sex, Lofgren syndrome, radiology stage, rs76740888, and rs78251590) that were statistically significant predictors of the binary dependent variable (disease prognosis). The variables with the highest *p*-values were removed from the model until all *p*-values for the remaining variables were ≤0.05. The logistic models calculated odds ratios (ORs) and their respective 95% confidence intervals (CIs)

## Results

### Functional Analyses of the Sarcoidosis-Related 439 Candidate Genes

WES, mutation site filtering, and annotation analyses of 116 sarcoidosis patients and 208 healthy controls revealed that 1,311 variants were significant and were allotted to 439 candidate genes (shown in **Figure 1**). We further performed GO analyses for these candidate genes filtered from the case–control Fisher’s exact test on Metascape.org (21). The most enriched GO terms were the epoxygenase P450 pathway [count = 5 (1.34%), log10(p) = −5.26], keratinization [count = 13 (3.48%), log10(p) = (−4.93)], and defective GALNT3 causing familial hyperphosphatemic tumoral calcinosis (HFTC) [count = 4 (1.07%), log10(p) = −4.19] (shown in **Supplementary Figure S2A**). The top-level Gene Ontology biological processes comprised metabolic process, developmental process, response to stimulus, and cell proliferation (shown in **Supplementary Figure S2B**). To capture the relationships among the terms, we analyzed the network of enriched terms where terms with a similarity >0.3 were connected by edges and selected the terms with the best *p*-value from every 20 clusters using Cytoscape on Metascape.org (shown in **Supplementary Figures S2C, D**).

Further KEGG pathway enrichment of 439 genes in the “cluster profiler” revealed four significant pathway aggregations, including “caffeine metabolism” (gene ratio: 3/90, adjusted *p*-value = 0.0073227), “drug metabolism—other enzymes’ (gene ratio: 6/90, adjusted *p*-value = 0.0073227), “retinol metabolism” (gene ratio: 6/90, adjusted *p*-value = 0.01552589), and “drug metabolism—cytochrome P450” (gene ratio: 6/90, adjusted *p*-value = 0.02368937; shown in **Supplementary Figures S3A, B**). In addition, a molecular complex detection (MCODE) analysis was performed to identify the modules within the protein–protein interaction (PPI) network (parameter degree cut-off ≥2 and the MCODE score ≥1.0) using Cytoscape software (22, 23). We found that 439 candidate genes were significantly clustered into three groups presented in green, red, and blue nodes. MCODE 1 (red nodes, MCODE score = 3.4) has 10 genes, i.e., CUL5, RBX1, EFTUD2, HSPA8, CAND1, RPA1, STAU1, ATAD3A, PABPC1, and SLC25A5. MCODE 2 (blue nodes, MCODE score = 1) contains RB1, ZNF99, and ZNF208. MCODE 3 (green nodes, MCODE score = 1) has three genes, including CYP2A7, CYP2F1, and CYP4F2 (shown in **Supplementary Figures S3C, D**).

In addition, the genes filtered by Fisher’s exact test with the genomic inflation factor are presented in **Supplementary Table S2**. The Manhattan plot (shown in **Supplementary Figure S4A**) showed visual identification of statistically significant data points with *p*<0.05. We tested for GO term (biological processes) enrichment to assess the gene-set covered biological functions and pathways. Four significantly enriched GO terms were detected, including loneliness (MATG) (p adjusted=0.000134505), loneliness (p adjusted=0.000742134), extremely high intelligence (p adjusted=0.032706257), and Plasminogen activator inhibitor type 1 levels (PAI-1) (p adjusted=0.038615263), see **Supplementary Figure S4B**.

### Selection and Functional Analysis of Immune-Related Genes

Previous studies suggested that sarcoidosis is an immune-related granulomatous disease associated with genetic susceptibility (24, 25). To distinguish the immune-related pathogenic genes in 439 candidate genes identified in our study, we searched the “Immune” term among “GO biological process” and found 36 immune-related GO terms (see **Table 1**) covering 135 variants of 30 immune-related genes. The SNP details of the 30 immune-related candidate genes are listed in **Supplementary Table S1**.

**Table 1:** Gene Ontology terms associated with “immune” in Cluster Profiler analyses.

GO ID	GO term	Count	Genes
GO:0002376	Immune system process	30	APOL1|AZGP1P1|BSG|CCL4|CNN2|COCH|CSF1R|CXCR4|EDN1|EZR|FLT1|GZMB|IL7|KCTD7|KIFAP3|KRT16P3|KRT6A|LILRA3|LILRA6|LILRB2|LILRB3|LILRB5|MARCH1|MSTN|NR1D1|PRSS3|RB1|RPA1|SAA1|SWAP70
GO:0006955	Immune response	17	APOL1|AZGP1P1|CCL4|COCH|CSF1R|EDN1|EZR|GZMB|IL7|KCTD7|KRT16P3|LILRB2|MARCH1|NR1D1|PRSS3|SAA1|SWAP70
GO:0002682	Regulation of immune system process	15	CCL4|COCH|CSF1R|EDN1|EZR|IL7|KCTD7|KRT6A|LILRA6|LILRB2|LILRB3|MSTN|NR1D1|RB1|SWAP70
GO:0045087	Innate immune response	10	APOL1|CCL4|COCH|CSF1R|EDN1|GZMB|KRT16P3|NR1D1|PRSS3|SAA1
GO:0002520	Immune system development	9	CNN2|CSF1R|IL7|LILRA6|LILRB2|LILRB3|RB1|RPA1|SWAP70
GO:0002684	Positive regulation of immune system process	9	CCL4|COCH|EDN1|EZR|IL7|KCTD7|LILRB2|NR1D1|SWAP70
GO:0002252	Immune effector process	5	GZMB|KCTD7|KRT6A|MSTN|SWAP70
GO:0050776	Regulation of immune response	5	COCH|EZR|KCTD7|LILRB2|NR1D1
GO:0002683	Negative regulation of immune system process	4	EZR|KCTD7|LILRB2|NR1D1
GO:0002764	Immune response-regulating signaling pathway	4	EZR|KCTD7|LILRB2|NR1D1
GO:0050778	Positive regulation of immune response	4	COCH|EZR|KCTD7|NR1D1
GO:0002697	Regulation of immune effector process	3	KCTD7|KRT6A|MSTN
GO:0002768	Immune-response-regulating cell surface receptor signaling pathway	3	EZR|KCTD7|LILRB2
GO:0002757	Immune-response-activating signal transduction	3	EZR|KCTD7|NR1D1
GO:0002253	Activation of immune response	3	EZR|KCTD7|NR1D1
GO:0002366	Leukocyte activation involved in immune response	2	KCTD7|SWAP70
GO:0002263	Cell activation involved in immune response	2	KCTD7|SWAP70
GO:0045089	Positive regulation of innate immune response	2	COCH|NR1D1
GO:0045088	Regulation of innate immune response	2	COCH|NR1D1
GO:0002429	Immune-response-activating cell surface receptor signaling pathway	2	EZR|KCTD7
GO:0002767	Immune-response-inhibiting cell surface receptor signaling pathway	1	LILRB2
GO:0002765	Immune-response-inhibiting signal transduction	1	LILRB2
GO:0002279	Mast cell activation involved in immune response	1	KCTD7
GO:0002312	B-cell activation involved in immune response	1	SWAP70
GO:0002562	Somatic diversification of immune receptors *via* germline recombination within a single locus	1	SWAP70
GO:0002200	Somatic diversification of immune receptors	1	SWAP70
GO:0050777	Negative regulation of immune response	1	LILRB2
GO:0002698	Negative regulation of immune effector process	1	KCTD7
GO:0002285	Lymphocyte activation involved in immune response	1	SWAP70
GO:0002699	Positive regulation of immune effector process	1	KCTD7
GO:0002758	Innate immune response-activating signal transduction	1	NR1D1
GO:0002218	Activation of innate immune response	1	NR1D1
GO:0016064	Immunoglobulin mediated immune response	1	SWAP70
GO:0002460	Adaptive immune response based on somatic recombination of immune receptors built from immunoglobulin superfamily domains	1	SWAP70
GO:0006959	Humoral immune response	1	IL7
GO:0002250	Adaptive immune response	1	SWAP70

**Figure 2:**
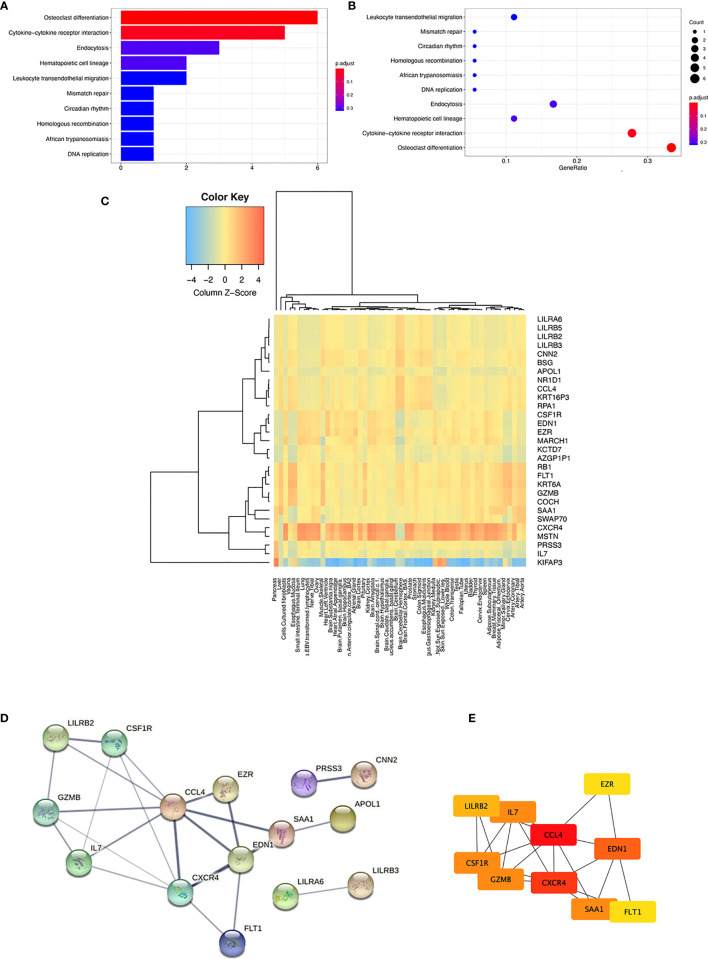
KEGG pathway analysis and expression characteristics of 30 immune-related candidate genes. **(A)** Bar plot of the KEGG pathway enrichment **(B)** Dot plot of the KEGG pathway enrichment **(C)** Organ and tissue expression characteristics analyzed by GTEx. A heatmap of the tissue-specific gene expression for 30 immune-related candidate genes in different organs and whole blood from the genotype-tissue expression project (GTEx) v8 54 tissue types of dataset. **(D)** PPI network of the 30 immune-related candidate genes and three modules were clustered by the STRING database. **(E)** PPI network of the top 10 hub genes of the 30 immune-related candidate genes.

Two significant KEGG pathways enrichments, “hsa04380: osteoclast differentiation” (p-adjust = 0.0000587, gene count = 6) and “hsa04060: cytokine–cytokine receptor interaction” (p-adjust = 0.019641002, gene count = 5), were revealed by analysis of the 30 immune-related genes. Six genes in “osteoclast differentiation” enrichment include CSF1R, LILRA3, LILRA6, LILRB2, LILRB3, and LILRB5. The genes involved in “cytokine–cytokine receptor interaction” included CCL4, CSF1R, CXCR4, FLT1, and IL7 (shown in **Figures 2A, B**). Immune-related candidate gene expression characteristics were also analyzed using genotype-tissue expression (GTEx) data to investigate the potential role of these genes in multiple organs and tissues. **Figure 2C** shows that CXCR4 and MSTN had the highest expression in the lung among the 30 immune-related candidate genes.

We further analyzed the PPI of 30 immune-related genes using the STRING database. When a “medium confidence = 0.400” was defined as the cutoff criterion of the minimum required interaction score, three clusters were identified from the PPI network (shown in **Figure 2D**). The largest cluster comprised 27 nodes and 24 edges, with an average node degree of 1.78 (PPI enrichment *p*-value = 2.27e−05). The hub genes were determined by overlapping the genes according to the top 10 nodes selected by the degree in cytoHubba (26). The identification of hub genes and module interactions is helpful in selecting the key genes that reveal the underlying molecular mechanisms of sarcoidosis pathogenesis-associated immune-related candidate genes (27). The top 10 hub genes were selected, and they were arranged by rank degree and presented in different colors (higher rank degree labeled red, lower rank degree marked yellow). The genes with the most significant rank were CCL4 and CXCR4, which had the most interrelation with other associated immune-associated genes (shown in **Figure 2E**).

### Three Missense Mutations Suggested Immune-Related Pathogenesis of Sarcoidosis

We next investigated non-synonymous SNPs, which have been thought to play a more critical role in pathogenesis, as they have different alleles encoding different amino acids. A total of nine non-synonymous SNPs were found in four immune-related genes (shown in **Table 2**). Among them, seven non-synonymous variants in three genes showed a significant difference in the frequency between 116 sarcoidosis and 208 healthy control groups, with much higher frequencies in the sarcoidosis group than in the control group (*p* < 0.001, odds ratio ≥1; see **Table 2**), including PRSS3, LILRA6 (LILRB3), and CNN2.

**Table 2:** Immune-associated non-synonymous variation details in the Fisher’s exact test.

Chr	Position	Chromosomal location	Ref	Alt	Variation ID	Frequency of case group	Frequency of control group	*p*-value	OR	Functional annotation	Gene detail	Exonic function
9	33796673	9p13.3	G	A	rs76740888	0.3147	0.01683	1.83E−28	26.83	Exonic	PRSS3	Non-synonymous
9	33797969	9p13.3	T	A	rs149664918	0.2241	0.05769	1.16E−09	4.719	Exonic	PRSS3	Non-synonymous
17	38253621	17q21.1	A	G	rs201066687	0	0.2428	4.58E−22	0	Exonic	NR1D1	Non-synonymous
19	54744710	19q13.42	C	T	rs1132600	0.1724	0.009615	4.27E−15	21.46	Exonic	LILRA6,LILRB3	Non-synonymous
19	54744711	19q13.42	C	G	rs1132599	0.1724	0.009615	4.27E−15	21.46	Exonic	LILRA6,LILRB3	Non-synonymous
19	54744722	19q13.42	T	C	rs1132597	0.181	0.009615	5.16E−16	22.77	Exonic	LILRA6,LILRB3	Non-synonymous
19	54745989	19q13.42	G	C	rs1052963	0.6336	0.3077	9.31E−16	3.891	Exonic	LILRA6,LILRB3	Non-synonymous
19	1037871	19p13.3	C	A	rs78251590	0.3621	0.06731	1.24E−20	7.865	Exonic	CNN2	Non-synonymous
19	1037640	19p13.3	C	T	rs200303627	0	0.1875	9.56E−17	0	Exonic	CNN2	Non-synonymous

Chr, chromosome; Ref, reference genome base type; Alt, alteration of sample base type; p-value, p-value of Fisher’s exact test between sarcoidosis case group and healthy control group; function annotation, region of mutation site annotation by refGene database; gene detail, annotation of transcripts related to mutation sites based on refGene database; exonic function, annotation of exome region from refGene database; AA change, annotation of amino acid changes of mutation sites based on refGene database.

**Figure 3:**
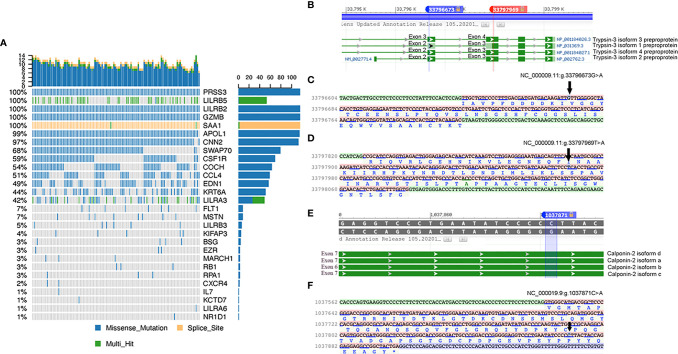
**(A)** Characteristics of SNPs of 30 immune-related candidate pathogenesis genes. The details of missense mutations, splice site mutations, and multihit mutations in 116 sporadic sarcoidosis patients are presented as percentages. **(B)** Two missense mutations in PRSS3 of transcript isoforms. **(C)** and **(D)** Mutation details of rs76740888 and rs149664918 on nucleotide sequence and amino acid sequence. **(E)** Missense mutation of rs78251590 in the CNN2 gene. **(F)** Mutation details of rs78251590 on nucleotide and amino acid sequences.

Furthermore, the mutation characteristics of 135 SNPs from the 30 immune-related candidate genes were analyzed using Maftools in R software. Among the three immune-related genes with significant mutation frequencies, the PRSS3 and CNN2 genes were detected, which contained the highest missense mutation ratios, at 100% and 97%, respectively, among the 116 sarcoidosis patients. Furthermore, non-synonymous SNPs in LILRB2, GZMB, APOL1, CNN2, SWAP70, and CSF1R were shown in over 50% of sarcoidosis patients. The SAA1 gene showed multiple hit and splice site mutations among 116 sporadic sarcoidosis patients (shown in **Figure 3A**). Two non-synonymous variants (NC_000009.11:g.33796673G>A and NC_000009.11:g.33797969T>A) in the PRSS3 gene were located on exon 3 of Trypsin-3 isoform 3 and exon 2 of Trypsin-3 isoforms 1, 2, and 4. A non-synonymous SNP in CNN2 (NC_000019.9: g.1037871C>A) was found in exon 7 of calponin-2 isoforms a, c, and d and exon 6 of calponin-2 isoform b (shown in **Figures 3B-F**).

### Univariate Analysis and Multivariate Logistic Regression Analysis Detected the Risk Factors for Disease Prognosis

This WES study included 58 (50%) acute sarcoidosis patients and 58 (50%) chronic prognosis sarcoidosis patients (shown in **Table 3**). Univariate analysis of the acute and chronic prognosis groups showed that the acute prognosis group was younger than the chronic prognosis group (*p*=0.016). Additionally, rs76740888 (G to A) and rs78251590 (C to A) mutations were associated with disease prognosis (*p*=0.034 and *p*<0.001, respectively).

**Table 3:** Univariate analysis of three missense mutations and clinical characteristics of 116 sarcoidosis patients.

Characteristic	Acute disease (<2 years)		Chronic disease (≥2 years)		*P*
No. of patients (%)	58	(%)	58	(%)	
Age					0.016^a^
<50 years	37	64%	24	41%	
≥50 years	21	36%	34	59%	
Gender					0.077^a^
Female	34	59%	43	74%	
Male	24	41%	15	26%	
Syndrome					
Lofgren syndrome	6		13		0.079^a^
Extrapulmonary involvement	8		8		1^a^
Laboratory tests					
ACE					0.343^a^
<68 (U/L)	49	84%	45	78%	
≥68 (U/L)	9	16%	13	22%	
ESR					0.709^a^
<15 (mm/h)	33	57%	31	53%	
≥15 (mm/h)	25	43%	27	47%	
hsCRP					0.576^a^
<3 (mg/L)	30	52%	33	57%	
≥3 (mg/L)	28	48%	25	43%	
Ca (mmol/L)					0.611^a^
<2.70 (mmol/L)	57	98%	55	95%	
≥2.70 (mmol/L)	1	2%	3	5%	
ALT (U/L)					0.488^a^
<40 (U/L)	55	95%	52	90%	
≥40 (U/L)	3	5%	6	10%	
NLR (X ± SD)	2.68 (1.92, 4.00)		2.57 (2.02, 3.35)		0.359^b^
BALF					
CD4/CD8 ratio					0.793^a^
<2.0	9	16%	8	14%	
≥2.0	49	84%	50	86%	
PFT					
FEV1/FVC (%)[M, (Q1, Q3)]	79.43 (75.59, 82.02)		78.95 (73.87, 84.29)		0.964^b^
DLCO (% pred)(X ± SD)	83.45 ± 13.22		84.34 ± 14.17		0.922^c^
CPI [M, (Q1, Q3)]	12.29 (2.21, 19.95)		14.8 (5.45, 23.41)		0.316^b^
Radiology stage^c^					0.09^a^
Stage I	19	33%	10	17%	
Stage II	30	52%	32	55%	
Stage III	9	16%	16	28%	
SNPs					
rs76740888					0.034^a^
GG	16		27		
GA	42		31		
rs149664918					0.709^a^
TT	33		31		
TA	25		27		
rs78251590					<0.001^a^
CC	44		14		
CA	14		44		

a *X* ^2^ test.

b Mann–Whitney U test.

c Independent samples t-test.

X ± SD, mean ± standard deviation; M, (Q1, Q3), median, first quartile, and the third quartile.

Radiology stage was according to the Scadding classification.

ACE, angiotensin converting enzyme; ESR, erythrocyte sedimentation rate; hsCRP, hypersensitive C-reactive protein; Ca, calcium in serum; ALT, alanine aminotransferase; NLR, neutrophil–lymphocyte ratio; BALF, bronchoalveolar lavage fluid; PFT, pulmonary function test; FEV1, forced expiratory volume in 1 s; FVC, forced vital capacity; DLCO, diffusing capacity of the lung for carbon monoxide for single-breath method; CPI, complex physiological index.

The related risk factors with *p*<0.1, including age, sex, Lofgren syndrome, radiology stage, rs76740888, and rs78251590, were set as the independent variables and included in multivariate logistic analyses. The disease prognosis was determined as the dependent variable. After adjustments for the founding variables, the binary logistic regression analysis showed that only age (*p*=0.037), radiology stage II (*p*=0.03), and rs76740888 (*p*=0.038) were retained as significant predictors of sarcoidosis prognosis. **Table 4** lists the variables and parameters that were finally screened into the model. Only age older than 50 (OR, 0.41), radiology stage II classification (OR, 0.25), and rs76740888 G to A mutation (OR, 2.49) were identified as independent factors correlated with an increase risk of chronic disease prognosis.

**Table 4:** Results of logistic regression analysis of risk factors for disease prognosis.

Variables	B	SE	Wald	*p*-value	OR value	95% CI for Exp(B)
Age (≥50)	−0.89	0.43	4.34	0.04	0.41	0.18−0.95
Sex (female)	−0.75	0.46	2.74	0.1	0.47	0.19–1.15
With Lofgren syndrome	−1.03	0.58	3.14	0.08	0.36	0.12–1.12
Radiology stage						
Stage I	–	–	–	–	Ref	
Stage II	−1.4	0.63	4.93	0.03	0.25	0.07–0.85
Stage III	−0.27	0.53	0.25	0.62	0.77	0.27–2.17
SNPs						
rs76740888						
GG	–	–	–	–	Ref	
GA	0.91	0.44	4.33	0.04	2.49	1.05–5.89

## Discussion

Sarcoidosis is an inflammatory disease characterized by granulomatosis present in multiple organs and triggered by environmental factors that interact with environmental triggers to result in the innate immune activation of macrophages and dendritic cells, which further upregulates the expression of the major histocompatibility complex (MHC) and cytokines that induce the activation of the adaptive immune response (3). Previous GWAS on the sporadic and familial aggregation of sarcoidosis patients showed that the candidate genes are strongly associated with disease severity, including HLA and non-HLA genes (9, 12). Most of the genes were related to T-cell regulation and T-cell activation during antigen presentation by APCs (antigen-presenting cells) (5). Other genes associated with immune regulation of sarcoidosis, including NOTCH4, TNFα, NOD2, and ANXA11, were also detected by GWAS analysis in different races (28–31). Multiple factors, including genetic composition and the context of antigen presentation, could impact the inflammatory immune response, resulting in a self-limiting or a chronic relapse type of sarcoidosis prognosis. The current study points out that the abundance of SNPs is associated with disease susceptibility and prognosis in sarcoidosis patients. A few papers have reported that human leukocyte antigen (HLA) DRB1*15 positivity is associated with an increased risk for a chronic course of sarcoidosis (32, 33). However, the susceptibility variants and the signaling pathways are different among different races of sarcoidosis patients (34–36). To our knowledge, this result is the first report on the genetics of Chinese sporadic sarcoidosis patients, which has significance for understanding immunogenetic pathogenesis and the development of chronic disease prognosis.

The present study revealed that 1,311 variants in 439 genes were present in 116 sporadic Chinese sarcoidosis patients compared to 208 healthy controls. Enrichment analysis with GO biological process terms revealed that 135 variants in 30 genes were related to the “Immune” associated GO term. The PRSS3 and CNN2 genes were detected with the highest missense mutation ratios (100% and 97%, respectively).

The PRSS3 (serine protease 3) gene product, trypsin-3, is a trypsinogen of the trypsin family of serine proteases and is expressed in multiple organs, such as the lung. The PRSS3 gene is located on the locus of T-cell receptor beta variable orphan on chromosome 9 ‘[cytogenetic location: 9p13.3; genomic coordinates (GRCh37/hg19) 33750677-33799229] and is associated with thyroiditis and Rickettsialpox (37, 38). This gene is localized to the locus of T-cell receptor beta variable orphans. It has been suggested to be involved in the proteolytic processing of proteins, digestion, blood coagulation, immune response, and development (39). Mesotrypsin/PRSS3 is an atypical isoform of trypsin that is expressed in the brain and other organs and is involved in the process of antimicrobial humoral response, cobalamin metabolic process, digestion, endothelial cell migration, neutrophil degranulation, proteolysis, and zymogen activation *via* calcium ion binding, protein binding, and serine-type endopeptidase activity signaling pathway (40). Pathways related to the PRSS3 gene on KEGG are “Influenza A,” “neuroactive ligand–receptor interaction,” “pancreatic secretion,” and “protein digestion and absorption” (41). Diseases associated with PRSS3 include thyroiditis and Hashimoto thyroiditis. The elevated expression of PRSS3 is associated with a poor prognosis for multiple cancers, including lung adenocarcinoma, gastric cancer, ductal carcinoma of the breast, and pancreatic cancer (37, 42–44). Two protein-coding SNPs on the PRSS3 gene identified in our research have not yet been published. The G to A missense mutation (rs76740888) in the exon of PRSS3 on chromosome 9p13.3 could cause an mRNA allele change and an amino acid change in four trypsin-3 isoforms, causing 10 coding sequence variants at each codon and amino acid. Protease imbalances have been found in another interstitial lung disease. Shanna Ashley et al. suggested that trypsin-3 was a potential biomarker for idiopathic pulmonary pneumonia (IPF) by proteomic analysis of plasma from IPF patients (45). How trypsin-3 influences sarcoidosis is still unknown.

CNN2 is located on chromosome 19p13.3 and functions as an actin cytoskeleton-associated protein and modifies the innate immune system pathways, including the inhibitory regulation of macrophage migration and phagocytosis (46). CNN2 is expressed in many organ tissues and cells, including epidermal keratinocytes, lung alveolar cells, and fibroblasts. In our study, we identified a C>A missense mutation (rs78251590) on CNN2 that may participate in the regulation of immune regulation of sarcoidosis.

We also attempted to identify biological pathways by inputting 30 candidate genes from the immune-related GO category. Two pathways were extracted from the KEGG analysis, including “hsa04380: osteoclast differentiation” (p-adjust = 0.0000587, gene count = 6) and “hsa04060: cytokine–cytokine receptor interaction” (p-adjust = 0.019641002, gene count = 5). A recent investigation of sarcoidosis illustrated that the differentially expressed genes (DEGs) identified by comparing the microarray datasets between sarcoidosis patients and healthy controls were significantly enriched in the positive regulation of protein kinase activity, osteoblast differentiation, and inflammatory response (47). The osteoclast differentiation and cytokine–cytokine receptor interaction pathways identified in our study may provide new ideas for understanding the role of immune-related gene pathways in Chinese sarcoidosis patients. Meanwhile, the “hsa04060: cytokine–cytokine receptor interaction’ pathway, including the CCL4, CSF1R, CXCR4, FLT1, and IL7 genes, could be highly associated with immune regulation and is strongly suspected to be involved in the pathogenesis of sarcoidosis (5, 48, 49). Interleukin (IL)-7 is essential for T-cell generation and plays a pivotal role in the proliferation and survival of memory and naive T cells and T helper type 17 (Th17) cells. Elliott Crouser et al. reported that IL-7 gene transcripts and transcript networks were highly engaged in pulmonary sarcoidosis biological processes and observed overexpression of the IL-7 protein in sarcoidosis patients. Similarly, Patterson et al. observed that the circulating cytokine IL-7 was increased in sarcoidosis patients compared to the control group (50). Keiichiro Yoshioka et al. used Gene Ontology enrichment analysis with RNA sequencing datasets. They revealed several biological processes related to the pathogenesis of sarcoidosis, such as cellular response to IL-1 and interferon gamma (IFN-γ), regulation of IL-6 production, and response to lipopolysaccharide. Meanwhile, they confirmed that the tumor necrosis factor (TNF), toll-like receptor signaling, and IL-17 signaling pathways were involved in the sarcoidosis pathobiology from KEGG pathway enrichment analysis (51).

The leading hub genes with variants are also essential regulators due to their changes in the activities of proteins and regulation mechanisms (47). Based on the analysis of the top 10 hub genes among 30 immune-related candidate genes, we found that CCL4 and CXCR4 were the most significant interrelated genes. C–C motif chemokine ligand 4 (CCL4) encodes a mitogen-inducible monokine involved in PEDF-induced signaling and the Akt signaling pathway, which could be secreted and involved in inflammatory functions. Barczyk et al. reported that the release of CCL4 chemokine was found to play a significant role in the recruitment of CD8+ T cells and CD4+ T cells to the inflammation sites in sarcoidosis patients (52). Another hub gene that we investigated was CXCR4, which encodes the C–X–C chemokine receptor type 4 protein and is characterized as the receptor for the C–X–C chemokine CXCL12/SDF-1 that transduces a signal by increasing intracellular calcium ion levels and contributes to enhancing MAPK1/MAPK3 activation. Katerina Antoniou et al. suggested that a significant increase in CXCR4 mRNA levels has been detected in sarcoidosis patients compared with healthy controls (53). CXCR4 has a functional relationship with sarcoidosis. The binding of bacterial lipopolysaccharide (LPS) mediates the LPS-induced inflammatory response and affects TNF secretion by monocytes, which are involved in excessive cytokine responses and induce the development of pulmonary sarcoidosis (54). LPS is mainly detected as a potential non-tuberculosis-associated pathogen-associated molecular pattern (PAMP) in sarcoidosis patients, which is an essential factor for the pathogenesis of sarcoidosis (55).

Interestingly, according to GO analysis of genes filtered from Fisher’s exact test with genomic inflation factor adjustment, we identified that the GO term “plasminogen activator inhibitor type 1 levels (PAI-1)” showed the highest proportion of overlapping genes in gene sets. PAI-1, also called “serpin family E member 1 (SERPINE1),” a member of the serine proteinase inhibitor (serpin) superfamily, has been shown to promote fibrosis in multiple organ systems and function as a component of innate antiviral immunity. Florence Jeny et al. identified that hypoxia increased the profibrotic response with PAI-1 secretion associated with human lung fibroblast migration inhibition in monocyte-derived (MD) macrophages among highly active sarcoidosis patients (56).

Finally, the correlation of genetic profiles with clinical outcomes through multivariate analysis showed that the missense variant (rs76740888, Chr9:33796673 G>A) of PRSS3 [*p*=0.04, odds ratio (OR)=2.49] was significantly associated with chronic prognosis. However, this candidate gene should be further analyzed to explore its potential and contribution to sarcoidosis prognosis. Furthermore, in keeping with prior reports, individuals with Stage II radiological classification had a more severe prognosis than those seen for the other stages. Manuel Rubio-Rivas and colleagues conducted a retrospective cohort study of 691 sarcoidosis patients. They suggested that stage II radiological classification at diagnosis was one of the risk factors related to the chronic trend of sarcoidosis (57).

Therefore, according to this study, the identified GO and KEGG pathways and immune candidate genes may act as pathogenesis and prognosis impactors for sarcoidosis. We acknowledge some limitations in our study. First, we need to validate the mechanisms that underlie the association between all genetic variants and sarcoidosis outcomes and the mediating pathway. Second, the findings need to be evaluated in larger cohorts before generalization due to the result being based on patients from a single center who developed sarcoidosis.

## Conclusion

Our WES study identified 135 SNPs in 30 candidate genes enriched in immune-related GO and KEGG pathways. Of these genes, we found that patients who carried missense mutations of rs76740888 (Chr9:33796673 G to A) on the PRSS3 gene had a higher probability of a chronic sarcoidosis prognosis. In addition, through a rigorous interrogation of candidate mutations in genes using available informatic data resources, we envisaged that the highly ranked hub genes among 30 immune-related candidate genes could also contribute to the pathogenesis of sarcoidosis, including CCL4 and CXCR4. Taken together, our data support the further understanding of the role of genetic mutations in immune regulation leading to the pathogenesis of sarcoidosis.

## Data Availability Statement

The data presented in the study are deposited in the NCBI repository, accession number PRJNA848857.

## Ethics Statement

The studies involving human participants were reviewed and approved by Bioethics Committee of the Medical University of Peking Union Medical College Hospital. The patients/participants provided their written informed consent to participate in this study.

## Author Contributions

QZ led the study at all stages and drafted the manuscript. CF, HH, and ZX designed the project and are involved at all stages. MZ and QZ designed and performed the data analyses. NW, WS, and XJ collected the clinical resources collection. JG and XY contributed to revising the manuscript. All authors contributed to the article and approved the submitted version.

## Funding

This study was funded by The National Natural Science Foundation of China (Grant 82070067) and The Beijing Municipal Natural Science Foundation (Grant 7212076).

## Conflict of Interest

Author MZ was employed by ANNOROAD Co.

The remaining authors declare that the research was conducted in the absence of any commercial or financial relationships that could be construed as a potential conflict of interest.

## Publisher’s Note

All claims expressed in this article are solely those of the authors and do not necessarily represent those of their affiliated organizations, or those of the publisher, the editors and the reviewers. Any product that may be evaluated in this article, or claim that may be made by its manufacturer, is not guaranteed or endorsed by the publisher.
