# Supplementary figures and images for: Exome Sequencing Reveals Genetic Variability and Identifies Chronic Prognostic Loci in Chinese Sarcoidosis Patients
Source: Front Oncol. 2022 Jul 4;12:910227. doi: 10.3389/fonc.2022.910227 (PMC9289133; doi:10.3389/fonc.2022.910227)

# HetReads Fraction Distribution

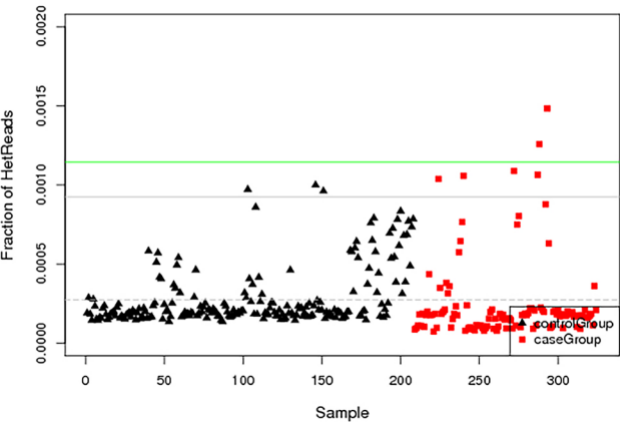

Supplement: Supplementary file 1 [file Image_1.pdf]

A

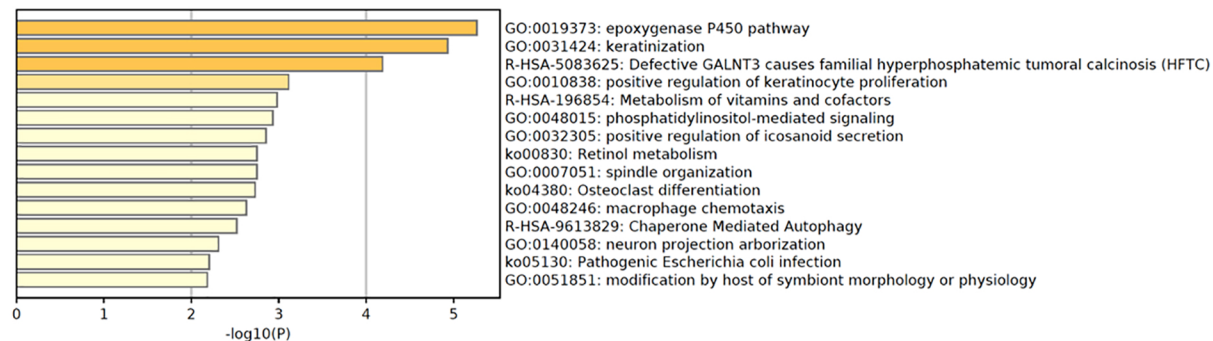

B

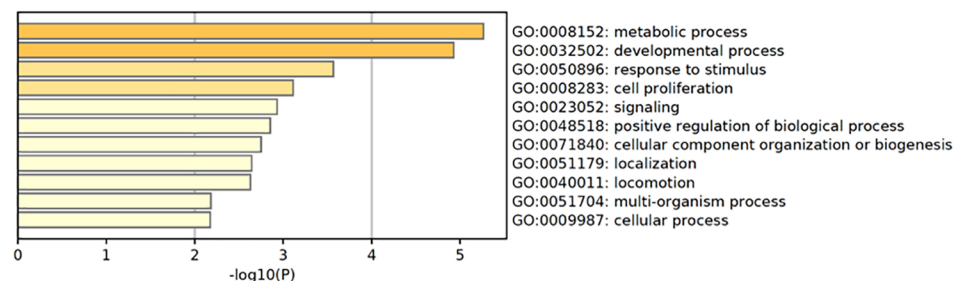

C

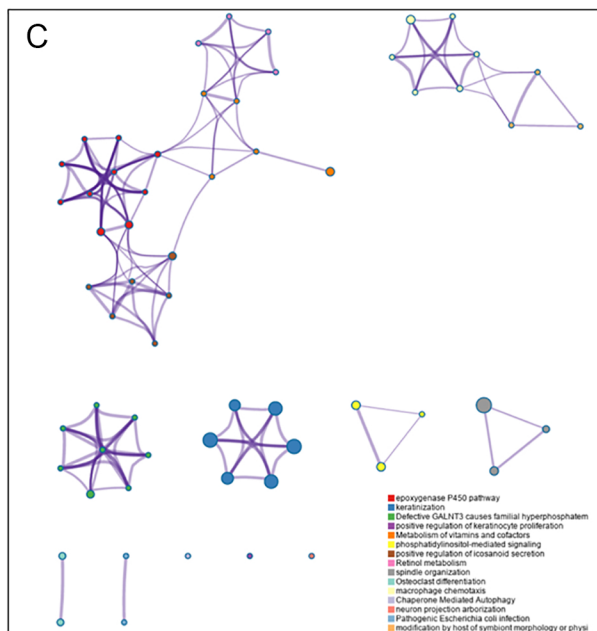

D

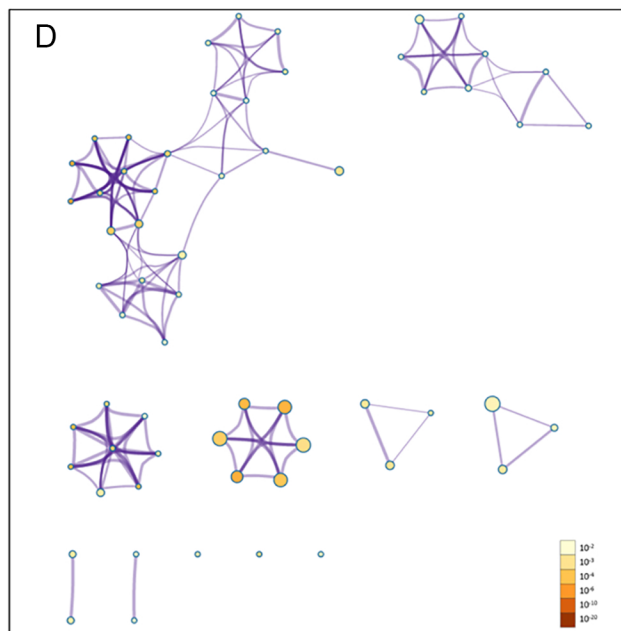

Supplement: Supplementary file 2 [file Image_2.pdf]

A

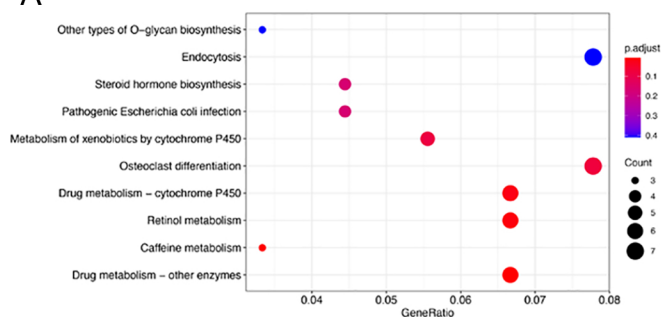

B

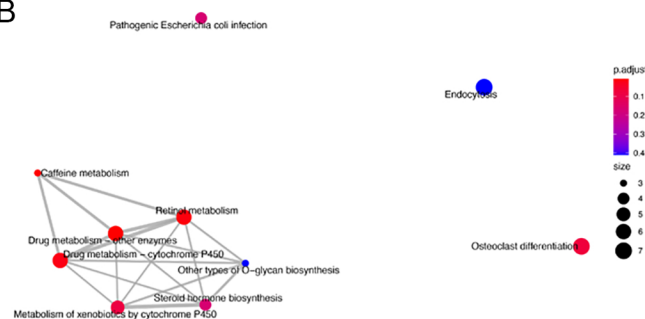

C

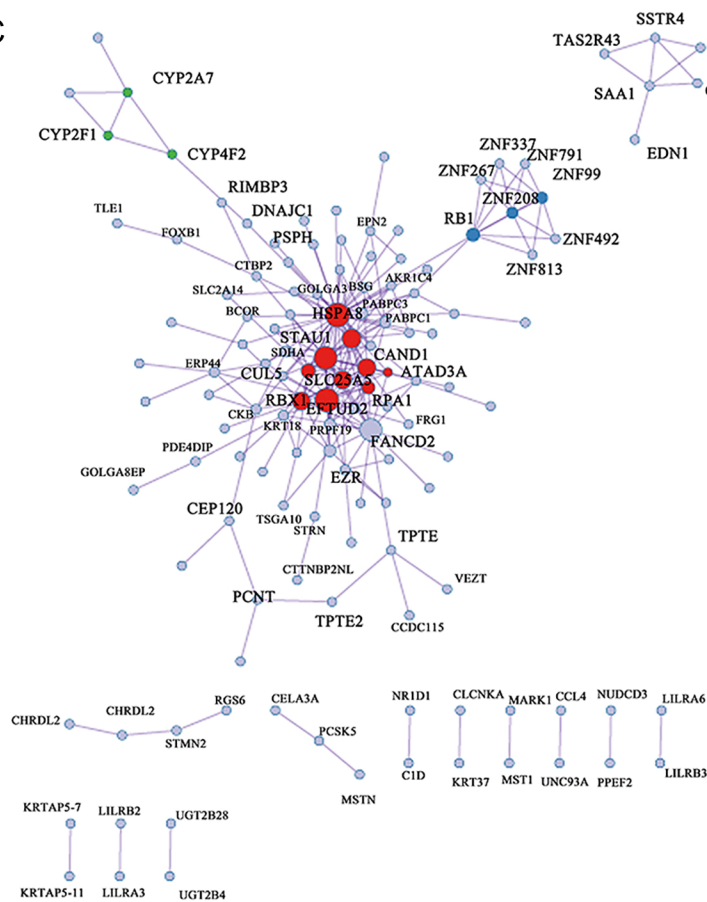

D

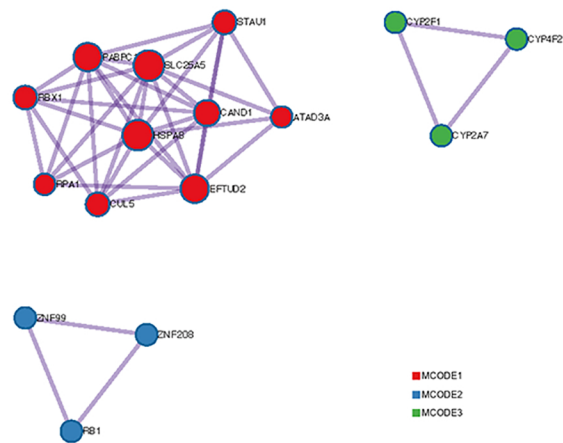

Supplement: Supplementary file 3 [file Image_3.pdf]

A

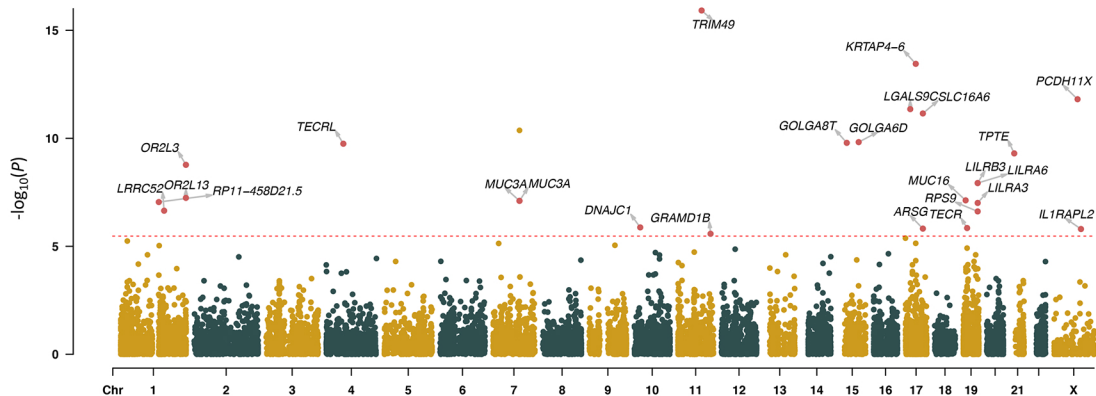

B

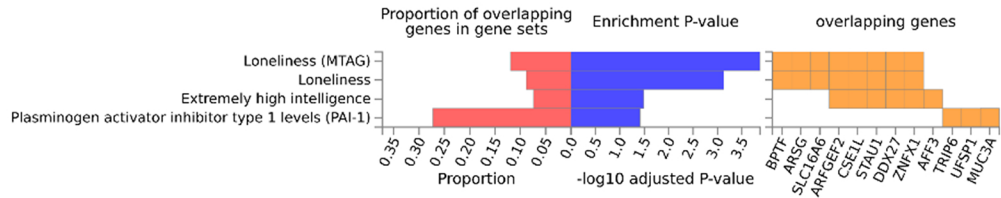

Supplement: Supplementary file 4 [file Image_4.pdf]
