# Supplementary material for: Exome Sequencing Reveals Genetic Variability and Identifies Chronic Prognostic Loci in Chinese Sarcoidosis Patients
Source: Front Oncol. 2022 Jul 4;12:910227. doi: 10.3389/fonc.2022.910227 (PMC9289133; doi:10.3389/fonc.2022.910227)
Supplement: Supplementary file 5 [file Table_1.pdf]

Supplementary Tables

Supplementary Table 1. SNP details of the 30 immune-related candidate genes.

Chr	SNP	Gene.refGene	Ref	Alt	Func.refGene	Exonic Function	P	OR
1	chr1:169941761	KIFAP3	A	T	intronic	.	5.67E-08	14.42
2	chr2:136958027	CXCR4,THSD7B	C	T	intergenic	.	5.18E-08	NA
2	chr2:190788887	PMS1,MSTN	A	G	intergenic	.	5.10E-33	50.18
2	chr2:190788928	PMS1,MSTN	G	C	intergenic	.	3.83E-14	35.55
2	chr2:190789106	PMS1,MSTN	C	T	intergenic	.	2.15E-08	15.15
2	chr2:190789109	PMS1,MSTN	G	A	intergenic	.	8.12E-09	15.88
2	chr2:190789116	PMS1,MSTN	C	T	intergenic	.	1.50E-08	12.44
4	chr4:164854211	MARCH1	T	C	intronic	.	2.05E-09	NA
4	chr4:164854220	MARCH1	C	T	intronic	.	7.87E-11	NA
4	chr4:164854238	MARCH1	G	A	intronic	.	6.93E-10	NA
4	chr4:164854268	MARCH1	T	C	intronic	.	3.24E-13	NA
4	chr4:164854274	MARCH1	A	G	intronic	.	1.07E-13	NA
4	chr4:164854289	MARCH1	T	C	intronic	.	3.24E-13	NA
4	chr4:164854295	MARCH1	T	C	intronic	.	3.24E-13	NA
4	chr4:164854298	MARCH1	A	C	intronic	.	3.24E-13	NA
4	chr4:164854300	MARCH1	C	A	intronic	.	3.24E-13	NA
4	chr4:164854320	MARCH1	T	C	intronic	.	8.83E-12	NA
4	chr4:164854324	MARCH1	G	C	intronic	.	8.83E-12	NA

4	chr4:164854329	MARCH1	T	G	intronic	.	8.83E-12	NA
4	chr4:164854342	MARCH1	C	T	intronic	.	2.64E-11	NA
4	chr4:164854348	MARCH1	C	T	intronic	.	2.64E-11	NA
4	chr4:164854356	MARCH1	C	A	intronic	.	7.87E-11	NA
5	chr5:149473946	CSF1R	C	T	intronic	.	4.16E-17	NA
5	chr5:149473952	CSF1R	A	G	intronic	.	3.99E-16	NA
5	chr5:149473966	CSF1R	A	G	intronic	.	3.99E-16	NA
5	chr5:149473980	CSF1R	A	T	intronic	.	3.99E-16	NA
5	chr5:149473981	CSF1R	A	G	intronic	.	3.99E-16	NA
5	chr5:149473986	CSF1R	T	C	intronic	.	3.99E-16	NA
5	chr5:149473987	CSF1R	G	A	intronic	.	3.99E-16	NA
5	chr5:149474011	CSF1R	T	C	intronic	.	3.77E-15	NA
5	chr5:149474020	CSF1R	A	G	intronic	.	9.78E-13	NA
5	chr5:149474023	CSF1R	A	G	intronic	.	3.24E-13	NA
5	chr5:149474044	CSF1R	A	G	intronic	.	2.94E-12	NA
5	chr5:149474047	CSF1R	T	C	intronic	.	2.94E-12	NA
5	chr5:149474060	CSF1R	A	C	intronic	.	8.83E-12	NA
5	chr5:149474062	CSF1R	G	C	intronic	.	8.83E-12	NA
5	chr5:149474068	CSF1R	A	G	intronic	.	8.83E-12	NA
5	chr5:149474074	CSF1R	A	G	intronic	.	2.64E-11	NA
5	chr5:149474080	CSF1R	G	C	intronic	.	2.64E-11	NA
5	chr5:149474092	CSF1R	A	G	intronic	.	2.34E-10	NA

6	chr6:12514446	EDN1,PHACTR1	T	G	intergenic	.	1.15E-14	NA
6	chr6:12514449	EDN1,PHACTR1	C	G	intergenic	.	1.15E-14	NA
6	chr6:12514488	EDN1,PHACTR1	C	A	intergenic	.	8.83E-12	NA
6	chr6:12514497	EDN1,PHACTR1	C	A	intergenic	.	2.34E-10	NA
6	chr6:12514515	EDN1,PHACTR1	T	C	intergenic	.	3.24E-13	NA
6	chr6:12514518	EDN1,PHACTR1	C	T	intergenic	.	3.24E-13	NA
6	chr6:12514521	EDN1,PHACTR1	T	C	intergenic	.	3.24E-13	NA
6	chr6:12514524	EDN1,PHACTR1	A	T	intergenic	.	9.78E-13	NA
6	chr6:12514527	EDN1,PHACTR1	T	C	intergenic	.	9.78E-13	NA
6	chr6:12514539	EDN1,PHACTR1	T	C	intergenic	.	2.94E-12	NA
6	chr6:12514551	EDN1,PHACTR1	C	T	intergenic	.	8.83E-12	NA
6	chr6:12514554	EDN1,PHACTR1	A	T	intergenic	.	8.83E-12	NA
6	chr6:12514599	EDN1,PHACTR1	T	C	intergenic	.	7.87E-11	NA
6	chr6:12514617	EDN1,PHACTR1	A	G	intergenic	.	2.94E-12	NA
6	chr6:12514620	EDN1,PHACTR1	G	A	intergenic	.	2.94E-12	NA
6	chr6:12514626	EDN1,PHACTR1	T	C	intergenic	.	2.94E-12	NA
6	chr6:12514647	EDN1,PHACTR1	A	T	intergenic	.	8.83E-12	NA
6	chr6:12514654	EDN1,PHACTR1	C	A	intergenic	.	8.83E-12	NA
6	chr6:12514658	EDN1,PHACTR1	T	C	intergenic	.	1.50E-10	47.88
6	chr6:159204426	EZR	T	A	intronic	.	1.40E-08	0.1624
7	chr7:99578575	AZGP1P1	C	T	ncRNA_intronic	.	2.95E-34	25.27
7	chr7:99578579	AZGP1P1	T	C	ncRNA_intronic	.	2.95E-34	25.27
7	chr7:66071830	GS1-124K5.11,KCTD7	G	T	intergenic	.	5.18E-08	NA

7	chr7:66071832	GS1-124K5.11,KCTD7	G	T	intergenic	.	5.18E-08	NA
7	chr7:66071833	GS1-124K5.11,KCTD7	T	A	intergenic	.	5.18E-08	NA
7	chr7:66071842	GS1-124K5.11,KCTD7	A	G	intergenic	.	1.77E-08	NA
7	chr7:66071845	GS1-124K5.11,KCTD7	C	T	intergenic	.	1.77E-08	NA
7	chr7:66071866	GS1-124K5.11,KCTD7	C	T	intergenic	.	5.18E-08	NA
8	chr8:80483983	IL7,STMN2	T	A	intergenic	.	6.93E-10	NA
8	chr8:80483995	IL7,STMN2	A	C	intergenic	.	2.64E-11	NA
8	chr8:80484000	IL7,STMN2	T	G	intergenic	.	8.83E-12	NA
8	chr8:80484004	IL7,STMN2	G	T	intergenic	.	8.83E-12	NA
8	chr8:80484016	IL7,STMN2	C	G	intergenic	.	6.93E-10	NA
8	chr8:80484039	IL7,STMN2	T	C	intergenic	.	5.18E-08	NA
8	chr8:80484040	IL7,STMN2	G	A	intergenic	.	5.18E-08	NA
9	chr9:33795512	PRSS3	T	C	intronic	.	1.44E-08	0
9	chr9:33795513	PRSS3	G	C	intronic	.	4.96E-08	0
9	chr9:33795516	PRSS3	T	C	intronic	.	4.96E-08	0
9	chr9:33796672	PRSS3	T	C	exonic	synonymous	1.83E-28	26.83
9	chr9:33796673	PRSS3	G	A	exonic	nonsynonymous	1.83E-28	26.83
9	chr9:33797726	PRSS3	C	A	intronic	.	3.42E-11	6.91
9	chr9:33797727	PRSS3	A	G	intronic	.	3.42E-11	6.91
9	chr9:33797749	PRSS3	C	G	intronic	.	2.15E-11	6.522

9	chr9:33797755	PRSS3	G	A	intronic	.	4.89E-11	6.351
9	chr9:33797767	PRSS3	G	T	intronic	.	9.36E-12	6.448
9	chr9:33797774	PRSS3	G	T	intronic	.	1.80E-10	5.963
9	chr9:33797896	PRSS3	G	A	exonic	synonymo us	2.87E-20	36.86
9	chr9:33797969	PRSS3	T	A	exonic	nonsynon ymous	1.16E-09	4.719
9	chr9:33799010	PRSS3	C	T	intronic	.	4.14E-16	19.24
9	chr9:33799398	PRSS3	T	A	downstream	.	5.18E-08	NA
11	chr11:18290902	SAA1	T	C	intronic	.	1.57E-10	0.3064
11	chr11:18290903	SAA1	T	C	intronic	.	9.76E-11	0.3034
11	chr11:18290906	SAA1	G	A	intronic	.	9.05E-11	0.3005
11	chr11:9627754	WEE1,SWAP70	A	G	intergenic	.	4.37E-12	0.06155
12	chr12:52885231	KRT6A	T	A	intronic	.	2.01E-08	3.629
13	chr13:48921884	RB1	A	G	intronic	.	6.93E-10	NA
13	chr13:48921884	RB1	A	G	intronic	.	6.93E-10	NA
14	chr14:31290730	SCFD1,COCH	T	C	intergenic	.	3.24E-13	NA
14	chr14:31290742	SCFD1,COCH	A	G	intergenic	.	1.15E-14	NA
14	chr14:31290745	SCFD1,COCH	T	C	intergenic	.	3.52E-14	NA
14	chr14:31290751	SCFD1,COCH	C	T	intergenic	.	3.52E-14	NA
14	chr14:25101440	GZMB	C	T	intronic	.	8.49E-11	4.886
17	chr17:34431884	CCL4	T	C	intronic	.	7.86E-15	0.06266
17	chr17:34431961	CCL4	T	C	exonic	synonymo us	1.86E-22	0.02276
17	chr17:20397312	LGALS9B,KRT16 P3	A	C	intergenic	.	3.63E-11	4.177

17	chr17:38253621	NR1D1	A	G	exonic	nonsynonymous	4.58E-22	0
17	chr17:1761436	RPA1	A	G	intronic	.	1.74E-35	31.17
17	chr17:1761445	RPA1	C	T	intronic	.	4.25E-38	37.71
17	chr17:1761447	RPA1	G	A	intronic	.	1.11E-39	44.9
17	chr17:1761457	RPA1	C	T	intronic	.	2.16E-41	47.38
17	chr17:1761502	RPA1	T	A	intronic	.	1.15E-12	7.344
17	chr17:1761504	RPA1	G	A	intronic	.	4.38E-13	7.889
17	chr17:1761548	RPA1	A	T	intronic	.	8.22E-36	194.4
17	chr17:1761570	RPA1	T	C	intronic	.	3.04E-43	NA
17	chr17:1761575	RPA1	C	G	intronic	.	7.97E-44	NA
17	chr17:1761580	RPA1	G	T	intronic	.	7.97E-44	NA
17	chr17:1761593	RPA1	G	A	intronic	.	6.10E-41	NA
19	chr19:580870	BSG	A	C	intronic	.	1.29E-16	NA
19	chr19:1037640	CNN2	C	T	exonic	nonsynonymous	9.56E-17	0
19	chr19:1037869	CNN2	C	T	exonic	synonymous	2.15E-22	8.015
19	chr19:1037871	CNN2	C	A	exonic	nonsynonymous	1.24E-20	7.865
19	chr19:54800222	LILRA3	A	G	intronic	.	9.27E-08	0.2278
19	chr19:54800225	LILRA3	A	G	intronic	.	9.27E-08	0.2278
19	chr19:54744710	LILRA6,LILRB3	C	T	exonic	nonsynonymous	4.27E-15	21.46
19	chr19:54744711	LILRA6,LILRB3	C	G	exonic	nonsynonymous	4.27E-15	21.46

19	chr19:54744722	LILRA6,LILRB3	T	C	exonic	nonsynonymous	5.16E-16	22.77
19	chr19:54744732	LILRA6,LILRB3	G	A	exonic	synonymous	1.65E-15	27.82
19	chr19:54745978	LILRA6,LILRB3	T	C	exonic	synonymous	1.89E-10	2.918
19	chr19:54745989	LILRA6,LILRB3	G	C	exonic	nonsynonymous	9.31E-16	3.891
19	chr19:54778822	LILRB2	G	A	intronic	.	7.43E-15	12.61
19	chr19:54778834	LILRB2	G	A	intronic	.	6.12E-12	8.102
19	chr19:54759571	LILRB5	T	C	intronic	.	3.79E-08	0.1083
22	chr22:36651325	APOL1	C	A	intronic	.	1.54E-51	320
22	chr22:36651333	APOL1	T	C	intronic	.	1.20E-53	177.2
22	chr22:36651340	APOL1	G	T	intronic	.	2.85E-55	355.2

Supplementary Table 2.The details of genes filtered from the Fisher exact test with genomic inflation factor

ensg	entrezID	symbol	hgnc_symbol	OMIM	uniprotID	DrugBank
ENSG00000132849	10207	INADL	INADL	603199	Q8NI35	NA
ENSG00000255168	NA	RP11-458D21.5	NA	NA	NA	NA
ENSG00000163386	100132406	NBPF10	NBPF10	614000	NA	NA
ENSG00000168509	148738	HFE2	HFE2	608374	Q6ZVN8	NA
ENSG00000117289	10628	TXNIP	TXNIP	606599	NA	NA
ENSG00000152022	128077	LIX1L	LIX1L	NA	NA	NA

ENSG00000198483	148741	ANKRD35	ANKRD35	NA	Q8N283	NA
ENSG00000162763	440699	LRRC52	LRRC52	615218	Q8N7C0	NA
ENSG00000196071	284521	OR2L13	OR2L13	NA	Q8N349	NA
ENSG00000198128	391192	OR2L3	OR2L3	NA	Q8NG85	NA
ENSG00000196240	401992	OR2T2	OR2T2	NA	Q6IF00	NA
ENSG00000196139	8644	AKR1C3	AKR1C3	603966	P42330	DB00157:DB00328:DB01536:DB01698:DB02056:DB02266:DB03461:DB07700
ENSG00000196326	340811	AKR1CL1	AKR1CL1	NA	NA	NA
ENSG00000198610	1109	AKR1C4	AKR1C4	600451	P17516	DB00157
ENSG00000173848	10276	NET1	NET1	606450	Q7Z628	NA
ENSG00000204682	399726	CASC10	CASC10	NA	Q5T4H9	NA
ENSG00000180592	387640	SKIDA1	SKIDA1	NA	NA	NA
ENSG00000078403	8028	MLLT10	MLLT10	602409	P55197	NA
ENSG00000136770	64215	DNAJC1	DNAJC1	611207	Q96KC8	NA
ENSG00000168930	57093	TRIM49	TRIM49	606124	P0CI25	NA
ENSG00000023171	57476	GRAMD1B	GRAMD1B	NA	Q3KR37	NA
ENSG00000166257	55800	SCN3B	SCN3B	608214	Q9NY72	DB00909
ENSG00000150048	51267	CLEC1A	CLEC1A	606782	Q8NC01	NA

ENSG00000139112	23710	GABARAPL1	GABARAPL1	607420	Q9H0R8	NA
ENSG00000134539	3824	KLRD1	KLRD1	602894	Q13241	NA
ENSG00000060140	55359	STYK1	STYK1	611433	Q6J9G0	NA
ENSG00000060138	8531	YBX3	YBX3	603437	P16989	NA
ENSG00000111215	11272	PRR4	PRR4	605359	Q16378	NA
ENSG00000212127	50840	TAS2R14	TAS2R14	604790	Q9NYV8	NA
ENSG00000212124	259294	TAS2R19	TAS2R19	613961	P59542	NA
ENSG00000256436	259290	TAS2R31	TAS2R31	612669	P59538	NA
ENSG00000268301	NA	AC018630.1	NA	NA	NA	NA
ENSG00000251655	5542	PRB1	PRB1	180989	NA	NA
ENSG00000121335	653247	PRB2	PRB2	168810	P02812	NA
ENSG00000102699	143	PARP4	PARP4	607519	Q9UKK3	NA
ENSG00000136156	9445	ITM2B	ITM2B	603904	Q9Y287	NA
ENSG00000139687	5925	RB1	RB1	614041	P06400	DB00030:DB00071
ENSG00000139679	10161	LPAR6	LPAR6	609239	P43657	NA
ENSG00000136161	1102	RCBTB2	RCBTB2	603524	O95199	NA
ENSG00000197168	341676	NEK5	NEK5	616731	Q6P3R8	DB12010

ENSG00000012963	55148	UBR7	UBR7	613816	Q8N806	NA
ENSG00000011114	55727	BTBD7	BTBD7	610386	Q9P203	NA
ENSG00000133958	57578	UNC79	UNC79	616884	Q9P2D8	NA
ENSG00000187581	341947	COX8C	COX8C	616855	Q7Z4L0	NA
ENSG00000261247	653075	GOLGA8T	GOLGA8T	NA	NA	NA
ENSG00000140478	653643	GOLGA6D	GOLGA6D	NA	P0CG33	NA
ENSG00000167202	23102	TBC1D2B	TBC1D2B	NA	Q9UPU7	NA
ENSG00000183476	646892	SH2D7	SH2D7	NA	A6NKC9	NA
ENSG00000136425	10518	CIB2	CIB2	605564	O75838	DB11093:DB11348:DB14481
ENSG00000166411	3419	IDH3A	IDH3A	601149	P50213	DB00157:DB06757:DB09092:DB09130
ENSG00000259511	100505679	UBE2Q2L	UBE2Q2L	NA	H0YL09	NA
ENSG00000224712	642778	NPIPA3	NPIPA3	NA	F8WFD2	NA
ENSG00000254852	642799	NPIPA2	NPIPA2	NA	E9PIF3	NA
ENSG00000103512	23420	NOMO1	NOMO1	609157	Q15155	DB00277
ENSG00000183426	9284	NPIPA1	NPIPA1	606406	Q9UND3	NA
ENSG00000179889	23042	PDXDC1	PDXDC1	614244	Q6P996	DB00114
ENSG00000157045	123803	NTAN1	NTAN1	615367	Q96AB6	NA

ENSG00000085721	54700	RRN3	RRN3	605121	Q9NYV6	NA
ENSG00000183793	100288332	NPIPA5	NPIPA5	606406	E9PKD4	NA
ENSG00000005187	6296	ACSM3	ACSM3	145505	Q53FZ2	NA
ENSG00000196678	112479	ERI2	ERI2	NA	A8K979	NA
ENSG00000005189	81691	AC004381.6	REXO5	NA	Q96IC2	NA
ENSG00000158486	55567	DNAH3	DNAH3	603334	Q8TD57	NA
ENSG00000103316	1428	CRYM	CRYM	123740	Q14894	DB05235
ENSG00000155719	146183	OTOA	OTOA	607038	Q7RTW8	NA
ENSG00000140740	7385	UQCRC2	UQCRC2	191329	P22695	DB04141:DB04799:DB07401:DB07763:DB07778:DB08330:DB08453:DB08690
ENSG00000155714	255762	PDZD9	PDZD9	NA	Q8IXQ8	NA
ENSG00000175267	146177	VWA3A	VWA3A	NA	A6NCI4	NA
ENSG00000103319	29904	EEF2K	EEF2K	606968	O00418	NA
ENSG00000103546	6530	SLC6A2	SLC6A2	163970	P23975	DB00182:DB00191:DB00193:DB00226:DB00234:DB00245:DB00285:DB00289:DB00321:DB00344:DB00408:DB00422:DB00454:DB00458:DB00476:DB00514:DB00540:DB00543:DB00579:DB00696:DB00715:DB00726:DB00830:DB00852:DB00907:DB00934:DB00937:DB00988:DB01105:DB01114:DB01142:DB01149:DB01151:DB01156:DB01170:DB01173:DB01175:DB01221:DB01242:DB01363:DB01364:DB01381:DB01442:DB01454:DB01576:DB01577:DB01579:DB04821:DB04836:DB04840:DB04889:DB04896:DB05012:DB05642:DB05688:DB05964:DB06148:DB06156:DB06204:DB06333:DB06700:DB06701:DB08918:DB09167:DB09185:DB09225:DB12305:DB14754
ENSG00000198848	1066	CES1	CES1	114835	P23141	DB00198:DB00382:DB00454:DB00583:DB00647:DB00907:DB01183:DB01452:DB01599:DB02161:DB02659:DB03056:DB0

						3721:DB04509:DB04795:DB04838:DB06442:DB07821:DB08224
ENSG00000181031	9501	RPH3AL	RPH3AL	604881	Q9UNE2	NA
ENSG00000171916	654346	LGALS9C	LGALS9C	NA	Q6DKI2	NA
ENSG00000034152	5606	MAP2K3	MAP2K3	602315	P46734	DB12010
ENSG00000263563	23666	UBBP4	UBBP4	NA	NA	NA
ENSG00000240871	100132476	KRTAP4-7	KRTAP4-7	NA	NA	NA
ENSG00000198090	81871	KRTAP4-6	KRTAP4-6	NA	Q9BYQ5	NA
ENSG00000198271	85289	KRTAP4-5	KRTAP4-5	NA	Q9BYR2	NA
ENSG00000175906	379	ARL4D	ARL4D	600732	P49703	NA
ENSG00000141198	10040	TOM1L1	TOM1L1	604701	O75674	NA
ENSG00000166260	1353	COX11	COX11	603648	Q9Y6N1	NA
ENSG00000166263	252983	STXBP4	STXBP4	610415	Q6ZWJ1	NA
ENSG00000171634	2186	BPTF	BPTF	601819	Q12830	NA
ENSG00000141337	22901	ARSG	ARSG	610008	Q96EG1	NA
ENSG00000108932	9120	SLC16A6	SLC16A6	603880	O15403	DB00119
ENSG00000070540	55062	WIPI1	WIPI1	609224	Q5MNZ9	NA
ENSG00000108946	5573	PRKAR1A	PRKAR1A	188830	P10644	DB01790:DB02315:DB02527:DB05798

ENSG00000141338	10351	ABCA8	ABCA8	612505	O94911	NA
ENSG00000181143	94025	MUC16	MUC16	606154	Q8WXI7	DB04964
ENSG00000188033	57474	ZNF490	ZNF490	NA	Q9ULM2	NA
ENSG00000269242	NA	CTD-2192J16.22	NA	NA	NA	NA
ENSG00000104774	4125	MAN2B1	MAN2B1	609458	O00754	NA
ENSG00000160961	84449	ZNF333	ZNF333	611811	Q96JL9	NA
ENSG00000127507	30817	EMR2	EMR2	606100	Q9UHX3	NA
ENSG00000197360	148198	ZNF98	ZNF98	603980	A6NK75	NA
ENSG00000229676	57615	ZNF492	ZNF492	NA	Q9P255	NA
ENSG00000213973	7652	ZNF99	ZNF99	603981	A8MXY4	NA
ENSG00000243130	5680	PSG11	PSG11	176401	Q9UQ72	NA
ENSG00000170889	6203	RPS9	RPS9	603631	P46781	DB11638
ENSG00000204577	11025	LILRB3	LILRB3	604820	O75022	NA
ENSG00000244482	79168	LILRA6	LILRA6	NA	Q6PI73	NA
ENSG00000105609	10990	LILRB5	LILRB5	604814	O75023	NA
ENSG00000131845	57343	ZNF304	ZNF304	613840	Q9HCX3	NA
ENSG00000268533	NA	AC004076.7	NA	NA	NA	NA

ENSG00000186230	388567	ZNF749	ZNF749	NA	O43361	NA
ENSG00000268163	NA	AC004076.9	NA	NA	NA	NA
ENSG00000178201	57191	VN1R1	VN1R1	605234	Q9GZP7	NA
ENSG00000197128	400720	ZNF772	ZNF772	NA	Q68DY9	NA
ENSG00000105136	79744	ZNF419	ZNF419	617410	Q96HQ0	NA
ENSG00000268107	NA	AC003005.4	NA	NA	NA	NA
ENSG00000152439	374928	ZNF773	ZNF773	NA	Q6PK81	NA
ENSG00000121406	256051	ZNF549	ZNF549	NA	Q6P9A3	NA
ENSG00000251369	162972	ZNF550	ZNF550	NA	Q7Z398	NA
ENSG00000083817	55659	ZNF416	ZNF416	NA	Q9BWM5	NA
ENSG00000171649	284307	ZIK1	ZIK1	NA	Q3SY52	NA
ENSG00000121417	10520	ZNF211	ZNF211	601856	Q13398	NA
ENSG00000197223	10438	C1D	C1D	606997	Q13901	NA
ENSG00000040933	3631	INPP4A	INPP4A	600916	Q96PE3	NA
ENSG00000196872	343990	KIAA1211L	KIAA1211L	NA	Q6NV74	NA
ENSG00000135951	80705	TSGA10	TSGA10	607166	Q9BZW7	NA
ENSG00000241962	150590	C2orf15	C2orf15	611838	NA	NA

ENSG00000273045	150590	C2ORF15	C2orf15	NA	Q8WU43	NA
ENSG00000144182	51601	LIPT1	LIPT1	610284	Q9Y234	DB00166
ENSG00000273155	51263	MRPL30	MRPL30	611838	NA	NA
ENSG00000158411	129531	MITD1	MITD1	NA	Q8WV92	NA
ENSG00000185414	51263	MRPL30	MRPL30	611838	Q8TCC3	NA
ENSG00000185674	254773	LYG2	LYG2	616547	Q86SG7	NA
ENSG00000144214	129530	LYG1	LYG1	NA	Q8N1E2	NA
ENSG00000158417	9669	EIF5B	EIF5B	606086	O60841	NA
ENSG00000144218	3899	AFF3	AFF3	601464	P51826	NA
ENSG00000155729	130535	KCTD18	KCTD18	NA	Q6PI47	NA
ENSG00000163535	151246	SGOL2	SGOL2	612425	Q562F6	NA
ENSG00000138356	316	AOX1	AOX1	602841	Q06278	DB03516
ENSG00000144451	79582	SPAG16	SPAG16	612173	Q8N0X2	NA
ENSG00000124198	10564	ARFGEF2	ARFGEF2	605371	Q9Y6D5	NA
ENSG00000124207	1434	CSE1L	CSE1L	601342	P55060	NA
ENSG00000124214	6780	STAU1	STAU1	601716	O95793	NA
ENSG00000124228	55661	DDX27	DDX27	616621	Q96GQ7	NA

ENSG00000124201	57169	ZNFX1	ZNFX1	NA	Q9P2E3	NA
ENSG00000158445	3745	KCNB1	KCNB1	600397	Q14721	DB06637
ENSG00000166157	7179	TPTE	TPTE	604336	NA	NA
ENSG00000138468	57337	SENP7	SENP7	612846	Q9BQF6	NA
ENSG00000176040	344805	TMPRSS7	TMPRSS7	NA	Q7RTY8	NA
ENSG00000114529	79669	C3orf52	C3orf52	611956	Q5BVD1	NA
ENSG00000174500	257144	GCSAM	GCSAM	607792	Q8N6F7	NA
ENSG00000172139	285335	SLC9C1	SLC9C1	612738	Q4G0N8	NA
ENSG00000131127	7700	ZNF141	ZNF141	194648	Q15928	NA
ENSG00000205678	253017	TECRL	TECRL	617242	Q5HYJ1	NA
ENSG00000156194	5470	PPEF2	PPEF2	602256	O14830	NA
ENSG00000138744	27163	NAAA	NAAA	607469	Q02083	NA
ENSG00000150627	116966	WDR17	WDR17	609005	Q8IZU2	NA
ENSG00000150628	132851	SPATA4	SPATA4	609879	Q8NEY3	NA
ENSG00000164674	94120	SYTL3	SYTL3	NA	Q4VX76	NA
ENSG00000092820	7430	EZR	EZR	123900	P15311	NA

ENSG00000146648	1956	EGFR	EGFR	131550	P00533	DB00002:DB00072:DB00281:DB00317:DB00530:DB01259:DB01269:DB03496:DB04988:DB05101:DB05294:DB05374:DB05424:DB05524:DB05944:DB06021:DB07602:DB07662:DB08916:DB09330:DB09559:DB10772:DB11731:DB11737:DB11828:DB11963:DB12010:DB12202:DB12267:DB13164
ENSG00000087077	7205	TRIP6	TRIP6	602933	Q15654	NA
ENSG00000176125	402682	UFSP1	UFSP1	611481	Q6NVU6	NA
ENSG00000169894	4584	MUC3A	MUC3A	158371	Q02505	NA
ENSG00000228273	4584	MUC3A	MUC3A	158371	NA	NA
ENSG00000187753	389766	C9orf153	C9orf153	NA	Q5TBE3	NA
ENSG00000135070	81689	ISCA1	ISCA1	611006	Q9BUE6	NA
ENSG00000083223	79670	ZCCHC6	ZCCHC6	613467	Q5VYS8	NA
ENSG00000175787	169841	ZNF169	ZNF169	603404	Q14929	NA
ENSG00000130950	54754	NUTM2F	NUTM2F	NA	A1L443	NA
ENSG00000148110	84641	HIATL1	HIATL1	NA	Q5SR56	NA
ENSG00000102290	27328	PCDH11X	PCDH11X	300246	Q9BZA7	NA
